# Videoconferencing analytic psychodrama in treating young adults’ psychological suffering: preliminary results

**DOI:** 10.3389/fpsyg.2023.1112711

**Published:** 2023-04-18

**Authors:** Roberta Biolcati, Federica Ambrosini, Alessandra Albani, Giovanni Di Stefano

**Affiliations:** ^1^Department of Education Studies “Giovanni Maria Bertin”, University of Bologna, Bologna, Italy; ^2^Department of Psychology, Educational Science and Human Movement, University of Palermo, Palermo, Italy

**Keywords:** alexithymia, analytic psychodrama, emotional intelligence, empathy, online psychotherapy, young adults

## Abstract

The study aims to explore the effects that videoconferencing Analytic Psychodrama (AP) has on the psychological wellbeing and emotional competence of young adults who are suffering from mental health problems. Twenty-two undergraduate students, asking for help at the Psychological Counselling Service of the University of Bologna for anxiety-depressive problems, took part in the three online groups that met weekly from October 2020 to July 2021. The Clinical Outcomes in Routine Evaluation Outcome Measure, the Trait Emotional Intelligence Questionnaire Short Form, the Toronto Alexithymia Scale, the Interpersonal Reactivity Index, and the Group Climate Questionnaire were used as test–retest questionnaires for clinical outcomes, emotional competence, and group climate evaluations. There was a statistically significant difference between the pre-test and 10-month scores for patients in clinical outcomes. Alexithymia significantly decreased and emotional intelligence and group engagement increased post-intervention. Videoconferencing AP sounds promising for alleviating psychological problems and to improve young adults’ emotional competence.

## 1. Introduction

Since the outbreak of COVID-19, the Psychological Counseling Service for Young Adults of the University of Bologna (Italy) adopted the solution to continue mental health care by providing interventions at a distance *via* videoconferencing psychotherapy. Online therapy is not a new practice in the mental health field, having been around for at least 20 years (Lambert et al., 2013), often practiced in specific situations, such as with patients living in isolated areas. Studies indicated that videoconferencing psychotherapy is feasible, has been used in a variety of therapeutic setting and with different psychological disorders, and has been found to have similar clinical outcomes to traditional face-to-face psychotherapy (Backhaus et al., 2012; Carrillo de Albornoz et al., 2022). Over recent years, research has highlighted the fact that online psychotherapy shows promising results for anxiety-depressive disorders (Berryhill et al., 2019 for a systematic review), and outcomes of internet interventions on mental health have become evidence-based (e.g., Karyotaki et al., 2018). Since the pandemic, the research and practice of online psychotherapy has increased exponentially (Weinberg, 2020). Despite the widespread use of online psychotherapy, studies on online group therapy remain still scarce (Weinberg, 2021), especially for psychoanalytically oriented group practices.

Among the individual and group treatments that the Counseling Service offered, Analytic Psychodrama (AP) is a model of group psychotherapy that is psychoanalytically oriented which recently documented evidence of efficacy (Biolcati et al., 2017; Orkibi and Feniger-Schaal, 2019). In the COVID-19 pandemic scenario, AP—considering the “body in action” as one of the core components of the intervention—proved to be quite challenging to convert online. Nevertheless, some psychotherapists trained in psychodrama working in the Counseling Service, through some technical adjustments, experienced the first videoconferencing sessions with their in-person groups to overcome the physical distancing of lockdown. From the favorable impression experienced in these first scenarios, the psychodramatists decided to propose three novel psychodrama groups entirely on videoconference to test their effects. This pilot study attempts to provide new insights and directions on the efficacy of online AP in clinical practice. A previous qualitative study (Biancalani et al., 2022) with a nonclinical group of adolescents found that tele-psychodrama was perceived by participants as helpful in improving their well-being in terms of self-awareness, self-confidence, relationships, and future perspective. However, to the best of our knowledge, this is the first study that aims to explore the effects of videoconferencing Analytic Psychodrama on the psychological wellbeing and the emotional competence of young adults who are suffering from anxiety-depressive disorders.

### 1.1. In-person analytic psychodrama

AP is based on a psychoanalytic perspective (Lemoine and Lemoine, 1973). This post-Freudian clinical model, while operating within Moreno’s role-playing tradition (Moreno and Rosati, 1985), is a different model from the Morenian one. Its first applications date back to 1956 in the treatment of children and adolescents with various problems (Anzieu, 1979). Later developments have indicated its efficacy on several psychopathological symptoms in young adults (e.g., Biolcati et al., 2017). AP aims to increase awareness about the unconscious mental contents from which suffering originates. At the same time, with the help of the group, it tends to create more functional patterns of behavior and communication. In the AP, patients’ repetition compulsion finds in the play and its enactment a basis for interrupting itself. Psychodramatic techniques include free association, role-play, role-reversal, doubling, a solo, and observation with feedback at the end of each session. In addition, the psychodynamic approach promotes attention to devices, such as dreams and lateral and vertical transference interpretations. An AP session begins with patients sitting in a circular configuration. The psychotherapist invites them to express what they are thinking, with free associations. From the narratives or dreams reported by the patients, the psychotherapist chooses a content (a real situation or a dream) that will be enacted, dramatized. The staging takes place right in the center of the patients’ circle. In addition to the protagonist of the scene, other members of the group, the so-called “auxiliary egos,” also participate in the staging. Their role is to play the role of significant others in the protagonist’s life (Karp and Farrall, 2014). During role-playing, the conductor may decide to interrupt the associative flow and reverse (one or more times) the roles of protagonists and auxiliary egos. Through the role-reversal technique, the protagonist has the opportunity to observe his/her own projections, to see his/her own relational scripts from the outside, and to understand the way he/she is seen by others. In this way, the protagonist has the chance to become familiar with his/her antagonist. In addition, during role-playing, the therapist and/or the patients can position themselves behind a character to voice feelings, thoughts, intentions, and needs that the protagonist may not be aware of or may not give voice to. This working technique, called “doubling,” when carried out by the therapist, takes on the meaning of a real analytic intervention. With a solo technique, the protagonist verbally expresses his or her thoughts and emotions after the role-playing. Psychodramatic play, symbolizing a real situation or a dream, brings out insights and new alternatives for thinking. Analytic psychodrama is a group psychotherapy that combines psychodramatic play with a psychoanalytical reading of “unconscious relational scripts.” Through role-playing, role-reversal, doubling, and a solo, psychodrama encourages getting in touch with emotions, whether it be one’s own or others’. If psychodrama works properly, in addition to improving symptomatology, the psychodramatist expects the patient to increase their ability to recognize and verbalize their emotions, increase their ability to put himself/herself in the other’s place, and to improve their ability to relate to others.

### 1.2. Analytic psychodrama working with young adults. Emotional intelligence, alexithymia, and interpersonal reactivity as target of intervention

During young adulthood, each individual has to face the delicate phase of transition from the family of origin and school context to the roles and responsibilities of adult life (Arnett, 2000; Masten and Cicchetti, 2010). This can sometimes result in a stressful condition that can precipitate the onset or recurrence of mental health problems (Blanco et al., 2008), such as anxiety and mood disorders, which are some of the most experienced problems in young adulthood (Pedrelli et al., 2015). University counseling services play a key role in preventing college students’ mental health problems from worsening, as they can readily take care of their psychological distress (Stallman, 2012). In this regard, from 1985 up to the present, the University of Bologna in Italy has offered a free counseling service for young adults, aimed at providing psychological support (Monti et al., 2013a,b).

Young adults need to stabilize emancipation from the family of origin and become protagonists in their own lives, improving the ability to regulate emotions and engage with significant others. Indeed, at this stage of life, although the individual’s responsibilities are increasing, the risk of poor psychosocial adjustment is greater because his or her emotional competence is still being consolidated (Hallam et al., 2014). The literature has shown that higher levels of social–emotional competence are associated with greater future well-being and lower risk of externalizing and internalizing problems (Bornstein et al., 2010; Jones et al., 2015). In itself, AP has demonstrated the potential to achieve positive change in group members and is considered an effective form of psychotherapy (Kipper and Ritchie, 2003). In addition, AP can be a useful treatment for dealing with difficulties peculiar to early adulthood. Recent evidence has shown the effectiveness of AP in reducing anxiety-depressive symptoms in young adults (Biolcati et al., 2017).

As it is psychoanalytically oriented, AP works on the underlying meanings of symptoms rather than focusing on symptoms themselves. In this way, it indirectly enables significant reduction in anxiety-depressive symptoms and personal distress (Biolcati et al., 2017). AP works with emotions, helping to recognize them appropriately in oneself and in others, and formulating the ability to put oneself in another’s shoes. The ultimate goal of psychodrama is not cathartic, but transformative. Group psychotherapy attempts to modify the profound aspects of the individual, such as certain ways of representing oneself and others. The change is more structural and concerns the most stable characteristics, such as the personality traits, of the patient and his/her way of “being in the world.” Starting with these theoretical considerations and driven by clinical practice, psychodramatists have chosen assessment tools based on “what they expected to change if the group well worked.”

#### 1.2.1. Trait emotional intelligence

Trait emotional intelligence (trait EI) addresses our perceptions of our emotional abilities; that is, how good we believe we are in terms of understanding, regulating, and expressing emotions in order to adapt to our environment and maintain well-being (Petrides et al., 2016). Trait EI emerges as an important individual difference variable and is composed of a constellation of emotional self-perceptions (often assessed through self-report questionnaires and rating scales) that constitute a person’s emotional attitude (Petrides et al., 2007). Several studies have investigated the influence of trait EI across the life span and, in particular, its impact on health (for a review, see Andrei et al., 2016), showing that trait EI is a strong positive predictor of well-being and mental health (Martins et al., 2010; Tolsa and Malas, 2022). Direct effects on general health were observed for trait EI in university students (Johnson et al., 2009). Moreover, low perceived EI has been inversely associated with anxiety and depression (Pettit et al., 2010; Shi et al., 2022).

#### 1.2.2. Alexithymia

While EI can be considered a protective factor for general health, alexithymia has, on the contrary, been identified as a potential risk factor related to mental health (Biolcati et al., 2021). Alexithymia (Nemiah and Sifneos, 1970) encompasses a cluster of cognitive and affective characteristics of which the main one is an inability to describe and/or recognize one’s own emotions (Brewer et al., 2015). Difficulty in identifying and describing feelings has been found to be negatively correlated with emotional well-being and overall health (Myles and Merlo, 2021). Furthermore, alexithymia was found to be indirectly associated with affective disorder symptoms *via* emotion regulation difficulties (Preece et al., 2022). The emotions of alexithymic individuals are relatively diffuse, poorly differentiated, and not psychically represented well (Naghavi et al., 2010). For example, in group psychotherapy settings, for several psychological problems (such as anxiety disorders and other mental disorders) alexithymia levels tend to decrease when clinical symptomatology improves (Fukunishi et al., 1997; Hemming et al., 2019).

#### 1.2.3. Empathy

Empathy is conceived as a multidimensional structure involving the perception, understanding, and sharing of others’ emotional states. It concerns the ability to look at events from one’s own perspective while putting oneself in the perspective of others in terms of thoughts, emotions, and behaviors (Dökmen, 2009). Empathic individuals tend to look at events from different points of view, put themselves in the other person’s perspective and resolve conflicts in a more positive way. Being the basis of communication, empathy is crucial in interpersonal relationships (Dogan, 2018). Young adults’ ability to resolve conflicts with empathy can have beneficial consequences for their well-being (Şimşek et al., 2020). Previous studies reported that empathy is positively related to mental health (Shanafelt et al., 2005) and quality of life in young adults, suggesting that empathy plays an important role in improving psychological well-being. However, the relationship between psychological well-being and different dimensions of empathy (i.e., perspective taking, fantasy, empathic concern and personal distress,) remains unclear. For example, empathic concern has been shown to be positively related to quality of life (Thomas et al., 2007), but not to mental health (Shanafelt et al., 2005); psychological well-being is positively predicted by perspective taking, but negatively predicted by personal distress.

#### 1.2.4. Group climate

Group climate (MacKenzie, 1983) is an indicator of the group’s atmosphere, and it was considered an important multidimensional construct. It includes participant’s perception of other members’ engagement with the group, avoidance of important issues or concerns, and conflict among group members (Gullo et al., 2015). A favorable group climate fosters the development of other positive therapeutic processes and, in general, is an important construct to review when evaluating the progress of group therapies (MacKenzie, 1998).

### 1.3. Aims and hypotheses

Starting from these premises, the present study aims to explore the effects that videoconferencing AP has on the psychological wellbeing and emotional competence of young adults who are suffering from anxiety-depressive problems.

Assuming that online psychodrama works as well as in-person psychodrama, at the end of 38 weekly sessions, the psychodramatists could expect:

1. a decrease in psychopathological symptoms;
2. a decrease in subjective distress;
3. an increase in trait EI (considered as a protective factor of psychological wellbeing);
4. a decrease in alexithymia (intended as a psychopathological risk factor);
5. an increase in empathy (intended as a positive interpersonal and pro-social capability); and
6. a more favorable group climate.

## 2. Method

### 2.1. Working method, frame and technical adjustments

Videoconferencing AP is an online, time-limited setting of group psychotherapy conducted by psychodramatists with a specialization in group psychotherapy at the Confederations of Italian Organizations for Analytic Research on Groups (COIRAG), and members of the Italian Society of Analytic Psychodrama (SIPsA).

The online setting consisted of weekly 90-min group sessions conducted on a web platform. (i.e., Zoom). The groups involved about seven to eight members of both genders and lasted 10 months. Each treatment included 38 sessions.

The setting was defined and shared with group members during the first session: privacy and confidentiality rules were stated, and agreement was reached about shared responsibility for the online setting.

The weekly online sessions began with free associations elicited by the psychotherapist. The “shared mental space” (necessary to bring out the symbolic games to be played) was characterized by being “all in the same place,” i.e., in the virtual room, reducing distractions as much as possible, with the help of the steady voice of the psychodramatist directing the session. Of course, in the role-reversal, the exchanges were only imaginative and verbal ones. During role-playing, the patient did not act as if he/she was in the other’s place but thought and spoke in place of the other without taking action. With the help of the therapists’ guidance, the imaginative role-playing forced group members to feel their emotions and transform them into words and symbolic play. Keeping the body still prevents the patients from being distracted from their emotions, unlike in acting, and makes the dialogue engaging. The imaginative ability to impersonate other roles allows the game of “as if.” The choice the protagonist makes about which members of the group will play the various characters in the scene has a symbolic meaning, which, for the analyst, is a valuable aspect to interpret.

Forgoing analytic abstinence to some extent, psychodramatists have been more direct in online sessions than in-person ones, to prevent distractions that might have been more insidious on the platform. Some useful guidelines included:

- Using Gallery view to see all of the patients who were hiding their self-view to avoid distractions;
- Clearly explaining the role-playing scripts, and avoiding confusion by introducing few characters and role-reversals;
- Encouraging doubling during and after the psychodramatic play to keep all participants within the “shared mental space”;
- Helping the participant to verbally express his or her emotions immediately after dramatizing a specific role;
- Speaking in a steady tone of voice to act as a supportive guide and transmit emotional closeness;
- Encouraging the sharing of dreams. The online platform precludes real movement, but the imagination, no longer restrained by the action of the body, generates dreams that are narrated;
- The way in which each participant uses technology can be a useful object of interpretation by the psychotherapist.

### 2.2. Design and procedure

The present study adopted the one-group pretest-posttest design. The study involved university students seeking help at the Counseling Service of the University of Bologna (Italy) (Vescovelli et al., 2017). After a four-session psychological consultation and a diagnostic assessment, the psychotherapist, supported by the staff, decided whether the patient needed further individual or group intervention. Patients who were referred for group psychotherapy and attended the three AP groups in videoconference from October 2020 to July 2021 were included in the study.

Ethical approval of this study was obtained from the Research Ethics Committees (REC) of the Department of Psychology of Bologna University. The patients/participants provided their written informed consent to participate in this study.

### 2.3. Participants

The original sample included 22 undergraduate students (13 females and 9 males) aged 20 to 28 years old (mean = 23.9, SD = 2.0), suffering from anxiety and depressive symptoms and seeking help at the Counseling Service of the University of Bologna (Italy). Since four patients dropped out before the end of the psychotherapy, the final sample was composed of 18 patients. Descriptive statistics of the final sample and the dropout group are shown in detail in Table 1.

**Table 1:** Comparison between completers and dropouts.

		Final sample (*n* = 18)	Dropouts (*n* = 4)	Comparison test	
Gender					
Males	*N* (%)	6 (67)	3 (25)	^a^2.35	*p* = 0.125
Females	*N* (%)	12 (33)	1 (75)		
Age					
	Mean (SD)	24.2 (1.9)	22.5 (2.1)	^b^−1.53	*p* = 0.141
Bachelor					
Bachelor of art	*N* (%)	13 (72.2)	4 (100.0)	^a^1.16	*p* = 0.281
Bachelor of science	*N* (%)	4 (22.2)	0 (0.0)		
Missing	*N* (%)	1 (5.6)	0 (0.0)		
ICD-9 Diagnosis N (%)					
300. Neurotic disorders	*N* (%)	7 (38.9)	2 (50.0)		
301. Personality disorders	*N* (%)	4 (22.2)	1 (25.0)	^a^1.12	*p* = 0.771
309. Adaptive reactions	*N* (%)	4 (22.2)	0 (0.0)		
313. Emotional disorders	*N* (%)	3 (16.7)	1 (25.0)		
CORE-OM					
Well-being	Mean (SD)	2.43 (0.74)	2.06 (0.63)	^b^0.09	*p* = 0.370
Problems	Mean (SD)	2.01 (0.74)	2.31 (0.42)	^b^0.77	*p* = 0.452
Functioning	Mean (SD)	1.86 (0.55)	2.13 (0.45)	^b^ 0.03	*p* = 382
Risk	Median (IQR)	2.05 (1.42–2.57)	2.23 (1.78–2.58)	^c^−0.384	*p* = 0.774
Total	Mean (SD)	1.69 (0.50)	1.86 (0.42)	^b^0.55	*p* = 0.530
Total-risk	Mean (SD)	2.00 (0.58)	2.20 (0.42)	^b^0.50	*p* = 0.547
TAS-20					
Total	Mean (SD)	52.33 (12.01)	56.75 (10.21)	^b^0.68	*p* = 0.505
TEIQue-SF					
Well-being	Mean (SD)	4.08 (1.02)	3.83 (0.98)	^b^−0.44	*p* = 662
Self-control	Mean (SD)	3.49 (0.93)	3.25 (1.36)	^b^−0.43	*p* = 0.670
Emotionality	Mean (SD)	4.73 (0.73)	4.44 (0.33)	^b^−0.77	*p* = 0.453
Sociability	Mean (SD)	3.78 (1.03)	3.92 (0.55)	^b^0.26	*p* = 0.798
Total Trait EI	Mean (SD)	4.01 (0.73)	3.89 (0.35)	^b^−0.31	*p* = 0.761
IRI					
Fantasy	Median (IQR)	25.50 (23.75–29.25)	27.00 (15.25–33.50)	^c^−0.214	*p* = 0.837
Perspective taking	Mean (SD)	26.83 (4.23)	27.00 (3.74)	^b^0.07	*p* = 0.943
Empathic concern	Mean (SD)	27.89 (3.50)	24.00 (3.92)	^b^−1.98	*p* = 0.062
Personal distress	Mean (SD)	24.44 (5.59)	21.75 (4.99)	^b^−0.89	*p* = 0.386
GCQ					
Engagement	Mean (SD)	20.17 (3.82)	22.00 (4.24)	^c^46.00	*p* = 0.404
Conflict	Mean (SD)	8.06 (2.65)	9.00 (2.83)	^b^64	*p* = 0.530
Avoidance	Mean (SD)	9.06 (2.82)	9.75 (1.71)	^b^0.47	*p* = 0.644

CORE-OM, Clinical Outcomes in Routine Evaluation-Outcome Measures; TAS-20, Toronto Alexithymia Scale; TEIQue-SF, Trait Emotional Intelligence Questionaire-Short Form; IRI, Interpersonal Reactivity Index; GCQ, Group Climate Questionnaire-Short Form. ^a^χ^2^-test; ^b^Independent samples *t*-test; ^c^Z standardized Mann–Whitney *U*-test value.

### 2.4. Tools

The Clinical Outcomes in Routine Evaluation Outcome Measure (CORE-OM; Evans et al., 2002), a self-report questionnaire designed to evaluate the effects of psychological therapies, was used to measure the baseline levels and outcomes of the online AP groups. The questionnaire is composed of 34 items with a 5-point Likert scale response (from “not at all” to “almost all the time”), assessing subjective Well-being (four items), Problems (12 items), Functioning (12 items), and Risk (four items for the risk of self-harm; two items for the risk of harm to others). A mean score ranging from 0 to 4 can be calculated for each domain, for the Total score, and for the Total score-risk items. Lower scores indicate better clinical outcomes. A clinical score ranging from 0 to 40 can be calculated in order to provide a classification of the symptoms/distress as “Severe” (≥25), “Moderate Severe” (≥20 – <25), “Moderate” (≥15 – <20), “Mild” (≥10 – <15), “Low Level” (≥6 – <10), and “Healthy” (0 – <6). In the present study, the CORE-OM showed good internal consistency, both in the first testing session and in the second testing session, for the total score subscale (Cronbach αpre = 0.88, Cronbach αpost = 0.81).

The Toronto Alexithymia Scale (TAS-20; Bagby et al., 1994) was used to assess alexithymia. It includes 20 items, rated from 1 (strongly disagree) to 5 (strongly agree) and evaluates the difficulty in identifying feelings (seven items), the difficulty in describing feelings (five items), and externally oriented thinking (eight items). A higher score implies worse levels of alexithymia. Scores that are ≤51 indicate no alexithymia, scores from 52 to 60 indicate possible alexithymia, and scores ≥61 indicate a condition of alexithymia. The TAS-20 internal consistency was good in both the first testing session (Cronbach α = 0.83) and in the second testing session (Cronbach α = 0.77).

The Trait Emotional Intelligence Questionnaire Short Form (TEIQue-SF; Petrides, 2009) was used to measure emotional intelligence. It is composed of 30 items with a 7-point Likert scale response (from 1 = Completely Disagree to 7 = Completely Agree). In addition to a global emotional intelligence mean score (ranging from 1 to 7), a mean score for each factor (Well-being, Self-control, Emotionality, and Sociability) can be obtained. The TEIQue-SF internal consistency was good for the Total Trait EI dimension (Cronbach αpre = 0.88, Cronbach αpost = 0.85).

The Interpersonal Reactivity Index (IRI; Davis, 1980) was used to measure the multifaceted aspects of empathy. Specifically, the tendency to strongly identify with fictional characters in books, movies, and plays (Fantasy subscale); the tendency to adopt the point of view of others (Perspective taking subscale); the other-oriented feelings of warmth, compassion, and concern (Empathic concern subscale); and the self-oriented feelings of discomfort and anxiety whilst witnessing the negative experiences of other people (Personal distress subscale). The tool is composed of 28 items, scored on a 5-point Likert scale ranging from “Does not describe me well” to “Describes me very well.” Four distinct total scores can be obtained for each of the IRI subscales. Internal reliability was acceptable for the Fantasy subscale (Cronbach αpre = 0.83, Cronbach αpost = 0.72) and the Personal distress subscale (Cronbach αpre =0.78, Cronbach αpost = 0.73). The Perspective taking subscale (Cronbach αpre = 0.69, Cronbach αpost = 0.54) and the Emphatic concern subscale (Cronbach αpre =0.57, Cronbach αpost = 0.52) showed a slightly lower internal reliability.

The Group Climate Questionnaire (GCQ; MacKenzie, 1983) was used to evaluate the group’s therapeutic environment according to the members’ perceptions. It included 12 items rated on a 7-point Likert scale (from “not at all” to “extremely”), which evaluates the Engagement (the perception of constructive therapeutic work, atmosphere, and group processes), the Avoidance (the tendency to avoid a constructive involvement in the group processes), and the Conflict (the perception of interpersonal anger, distancing, distrust, and tension within the group). A total score for each subscale can be calculated. In the present study, Cronbach alpha levels of the first and second tests for the subscales of the GCQ were 0.78 and 0.72 for Engagement; 0.16 and 0.55 for Avoidance; and 0.57 and 0.37 for Conflict.

### 2.5. Statistical analysis

Statistical analysis was performed using SPSS 26.0 (IBM Corp, 2019). Descriptive statistics were calculated. The normality assumption was verified through the visual inspection of histograms and Q-Q plots, and the Shapiro–Wilk test. As four individuals dropped out, after having verified the homogeneity of variances with the Levene’s test, a set of inferential statistics were performed to compare the completer’s group and the dropout’s group based on gender, age, kind of bachelor, diagnosis, and the baseline levels of CORE-OM, TAS, TEIQue-SF, IRI, and GCQ. Specifically, a chi-square test was used to compare the groups based on the categorical variables (gender, kind of bachelor, and diagnosis); independent-sample *t*-test was used to compare the groups based on the quantitative and normally distributed variables (age, CORE-OM mean scores of well-being, problems, functioning, total score, and total score-risk; TAS-20 total score; all TEIQue-SF factors mean scores; all IRI subscales scores; and GCQ Avoidance and Conflict), whereas the Mann–Whitney *U*-test was used to compare the groups based on quantitative and non-normally distributed variables (CORE-OM risk; IRI Fantasy).

Based on the variables’ distribution, a set of paired-sample t-test and Wilcoxon Rank sum test was used to compare pre- and post-scores at CORE-OM, TAS-20, TEIQue-SF, IRI, and GCQ. Within these analyses, only the scores of participants who completed all of the online AP group sessions were included (*N* = 18).

## 3. Results

The final sample included 12 females and six males aged 21–28 years old who completed all the psychodrama group sessions. As can be observed in Table 1, no statistically significant differences between the dropouts’ group and the completer’s group were found in age, gender, type of bachelor, and diagnosis. Moreover, no significant differences between the dropouts’ group and the completer’s group were found in the baseline levels of CORE-OM, TAS-20, TEIQue–SF, IRI, and GCQ.

**Figure 1:**
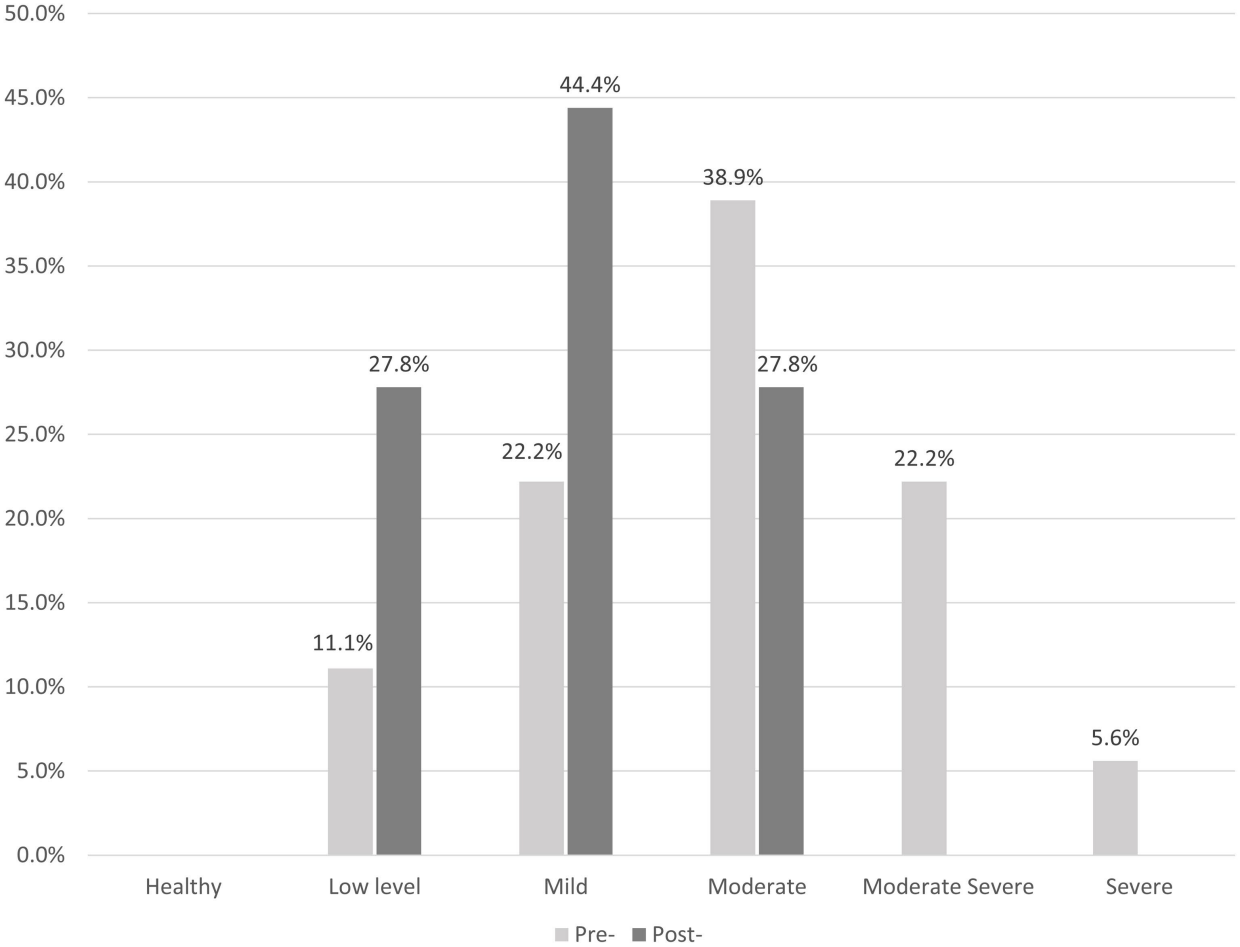
Pre- and post-intervention levels of symptoms and distress, based on CORE-OM clinical cut-offs.

As can be observed in Table 2, significant differences between the pre- and post-evaluation scores were found in all of the domains’ CORE-OM mean scores, except for the Risk subscale, thereby showing an improvement in levels of Well-being and Functioning, a decrease in the level of problems, and a general improvement in both the total score and the total score-risk subscales. Figure 1 displays the overall levels of symptoms and distress before and after the intervention, according to the CORE-OM’s clinical cut-off.

**Table 2:** *T*-test and Wilcoxon signed-rank test results of pre- and post-videoconferencing analytic psychodrama groups.

		Pre	Post	Statistic	*p* value
CORE-OM					
Well-being	Mean (SD)	2.43 (0.74)	1.72 (0.52)	^a^3.80	**0.001**
Problems	Mean (SD)	2.01 (0.74)	1.40 (0.58)	^a^4.98	**<0.001**
Functioning	Mean (SD)	1.86 (0.55)	1.35 (0.42)	^a^4.21	**0.001**
Risk	Median (IQR)	2.05 (1.42–2.57)	1.36 (1.12–1.76)	^b^−1.61	0.107
Total	Mean (SD)	1.69 (0.50)	1.19 (0.35)	^a^5.94	**<0.001**
Total-Risk	Mean (SD)	2.01 (0.58)	1.43 (0.41)	^a^5.93	**<0.001**
TAS-20					
Total	Mean (SD)	52.33 (12.01)	47.11 (10.22)	^a^3.15	**0.006**
TEIQue-SF					
Well-being	Mean (SD)	4.08 (1.02)	4.44 (0.79)	^a^−2.26	**0.038**
Self-control	Mean (SD)	3.49 (0.93)	3.60 (0.82)	^a^−0.71	0.486
Emotionality	Mean (SD)	4.73 (0.73)	5.01 (0.79)	^a^−2.48	**0.024**
Sociability	Mean (SD)	3.78 (1.03)	3.81 (0.82)	^a^−0.30	0.770
Total Trait EI	Mean (SD)	4.01 (0.73)	4.15 (0.68)	^a^−2.78	**0.013**
IRI					
Fantasy	Median (IQR)	25.50 (23.75–29.25)	28.00 (26.00–29.25)	^b^−2.36	**0.018**
Perspective taking	Mean (SD)	26.83 (4.23)	27.72 (2.82)	^a^−1.23	0.235
Empathic concern	Mean (SD)	27.89 (3.50)	27.39 (3.40)	^a^0.59	0.560
Personal distress	Mean (SD)	24.44 (5.59)	23.00 (4.92)	^a^1.85	0.082
GCQ					
Engagement	Mean (SD)	20.17 (3.82)	25.06 (2.98)	^a^−6.14	**<0.001**
Conflict	Mean (SD)	8.06 (2.65)	6.56 (2.12)	^a^3.17	**0.006**
Avoidance	Mean (SD)	9.06 (2.82)	8.83 (2.94)	^a^42	0.679

CORE-OM, Clinical Outcomes in Routine Evaluation-Outcome Measures; TAS-20, Toronto Alexithymia Scale; TEIQue-SF, Trait Emotional Intelligence Questionaire-Short Form; IRI, Interpersonal Reactivity Index; GCQ, Group Climate Questionnaire-Short; ^a^paired sample *t*-test, with 17 df; ^b^*Z* standardized Wilcoxon signed-rank value. Bold values indicate *p* < 0.05, two-tailed.

Levels of alexithymia, as measured by the total scores at TAS-20, significantly decreased. Considering clinical cut-offs, before the intervention, 28% of patients showed alexithymia, 28% showed possible alexithymia, and 44% did not show alexithymia. After the videoconferencing AP groups, only 6% of patients showed alexithymia, 33% showed possible alexithymia, and 61% did not show alexithymia.

For that which concerns the Trait EI, the total score at the TEIQue-SF was found significantly higher at the final evaluation. Moreover, a significant increase was observed in the Well-being and Emotionality factors. No significant differences between the pre- and post-scores emerged in the Self-control and Sociability factors.

For that which concerns empathy, there were significant differences between the scores of the first and final evaluations in the IRI Fantasy subscale, but not in Perspective taking, Empathic concern, and Personal distress subscales.

Lastly, regarding the perception of the therapeutic environment as measured by GCQ, we observed at the final evaluation a significant increase in the Engagement subscale and a significant decrease in the Conflict subscale. No significant differences were found between the pre- and post-scores of the Avoidance subscale.

## 4. Discussion

The pilot study investigating the effects of videoconferencing AP on the psychological well-being, emotional intelligence, alexithymia, and empathy of young adults with psychological disorders provided encouraging results. Our findings showed a significant improvement in patients’ levels of Well-being and Functioning, and a decrease in the Symptoms domain. The study results reinforce research that has found video-based groups to be feasible and have resulted in outcomes similar to in-person groups (Biolcati et al., 2017; Gentry et al., 2019; Weinberg, 2020). Additionally, levels of alexithymia significantly decreased and the Total Trait EI was found significantly higher at the final evaluation. Then, the Fantasy dimension of empathy and group Engagement increased, with a significant decrease in group Conflict.

Even if many psychodynamic psychotherapists considered online therapy “sacrilegious” before the pandemic (e.g., Essig, 2020) and thought it was not a type of “real therapy,” many of these individuals have since adjusted to the new situation, understanding that videoconferencing group psychotherapies are now a necessary fact needing research and evaluation.

Several considerations arose from this experience. From the point of view of clinical practice, many obstacles, and doubts about the online adaptation of the method of work remained; in the transition from the “physical circle” to the screen, the impossibility of bodily interaction in online groups can be regarded as the main obstacle, as previously observed by Biancalani et al. (2022). The therapist must make a greater effort to establish a strong therapeutic alliance, boosting group cohesion and developing an online presence that can overcome the lack of body-to-body interaction. Role-playing sometimes risks being “artificial” if one does not immerse oneself in the online space, making sure to avoid distractions and make an important mental effort to identify and empathize with the roles played. Inevitably, the meanings of the relationships of proximity to distance of the bodies in the dramatization and much of the non-verbal behaviors are also lost. In contrast, facial expressions become very important as does tone of voice. As Weinberg suggested (2020), to increase the therapist’s presence in online settings, he/she can make greater use of imagination, inviting group members to do the same. Specific training for improving a therapist’s self-confidence to conduct online group therapy is recommended.

As far as the empirical results, the findings are encouraging and inviting to persevere in future research. At the present time, there are no research data on the same constructs—namely EI, alexithymia, and empathy—for comparison between online psychodrama and in-person psychodrama outcomes. However, the findings of the present study suggest a reduction in symptoms and improved functioning for patients, even in the absence of bodily and physical proximity.

In addition to improving well-being, more structural factors such as Trait EI and alexithymia also changed after the online treatment. The findings showed a significant improvement in Well-being, Emotionality, and Total EI after intervention. The findings of this study show similarities with research that has addressed the issue of enhancing Trait EI through psychological interventions or creative programs (Ruttledge and Petrides, 2012; Tiabashvili et al., 2018; Sambol et al., 2022). Moreover, a recent overview of the literature (see Hemming et al., 2019) suggests that therapeutic interventions can partially modify alexithymia. In particular, interventions focused on alexithymic symptoms tend to show significant reductions in alexithymia scores when compared with psychological interventions not focused specifically on alexithymia, which show more inconsistent results. Our results suggest that videoconferencing AP and working with emotions decreases the alexithymic trait in young adults and increases EI.

Interestingly, Fantasy is the only dimension of empathy that changed in a statistically significant degree after treatment. There has been growing acceptance of the view that empathy can best be considered a multidimensional phenomenon encompassing both cognitive and affective elements (Davis et al., 1994; Davis, 2004). When using the IRI questionnaire, many scholars combine Perspective taking and Fantasy as “cognitive empathy” and empathic concern and Personal distress as “affective empathy” (Yan et al., 2021). Unlike expected, Perspective taking - the ability to adopt another’s perspective or point of view – although increasing has not significantly changed. Instead, the only significant change concerns Fantasy - the scale more difficult to characterize (Gilet et al., 2013) referring to the propensity to identify with fictitious situations, such as characters from books, movies or plays. The Fantasy dimension measures people’s ability to be imaginatively transported by fictional material. This makes us hypothesize that the screen could make the dramatizations live at a more imaginary level (like in a movie) than in real life and this could be a specific effect of being online. Indeed, it is not excluded that the two-dimensional screen leads to increase of the person’s empathic ability to identify with virtual characters as if they were not real. But these are inferences to be further investigated in future studies. However, previous research (Nomura and Akai, 2012) indicated that empathic processes addressed to real and fictional characters are similar and, therefore, suggested revising the IRI’s fantasy subscale, which is restricted to fictional situations. Additionally, the non-significant increase in affective empathy post-treatment may not be a negative sign, as it might sometimes constitute one of the risk factors for depression, and higher affective empathy might mean more vulnerability to depression (Yan et al., 2021).

As far as group climate, the Engagement is one dimension that increased significantly in our sample. It measures the sense of closeness, group members´ attempts to understand the meaning of behavior, the importance of the group for its members, a willingness to challenge one another, and self-disclosure (MacKenzie, 1983). This group-level process has been predictive of successful group and individual outcomes (Strauss et al., 2008; Arrow et al., 2021).

## 5. Limits and conclusion

Research on videoconferencing group therapy is still relatively young and lacks clear theoretical and practical guidelines for determining its efficacy. First, control group design with random assignment is needed to confirm the favorable impression of videoconferencing AP effectiveness in treating young adults’ suffering. Secondly, the small number of patients and the lack of a long-term follow-up allowed only an initial exploration of the topic and so conclusions should be handled with caution. Future lines of research should also investigate the differences in outcomes between online and in-person psychodrama by using the same constructs to better discriminate the effects that body presence has on emotional competence and clinical outcomes. Finally, the reliability of some scales such as group climate is quite low. The low reliability of the group climate subscales is reported in several studies; nevertheless, the literature suggests including findings of these measures to make comparisons to a large body of research that has used the GCQ (Bìlìcan and Mceneaney, 2018).

Despite the aforementioned limits, online psychodrama is a useful resource that young adults could draw on, not only in an emergency, but also in other circumstances during which they need psychological care. Studies suggest that online psychotherapy might also be advantageous for individuals who have difficulty moving or suffer economic constraints, and/or for patients in acute crisis with no access to in-person encounters (Biancalani et al., 2021). In addition, young people have a lot of experience with electronic devices and utilizing the Internet, and the videoconferencing psychotherapy could be an opportunity for expression and sharing of feelings, emotions, and suffering behaviors. Indeed, some patients, such as those with social phobia, may benefit from the videoconferencing setting, as in this context, they may find it easier to manifest openness and talk about their fears, concerns, and feelings. Online psychotherapy, therefore, could be particularly useful for young adults with high levels of social anxiety (Yen et al., 2012) and depression (Lira and Martínez, 2022).

In conclusion, this study contributed to extant literature on the positive effects of online AP for young adults on psychological symptoms, functioning, well-being, and emotional competence; furthermore, it adds new insight to studies on psychological interventions on the Internet.

## Data availability statement

The raw data supporting the conclusions of this article will be made available by the authors, without undue reservation.

## Ethics statement

Ethical approval of this study was obtained from the Research Ethics Committees (REC) of the Department of Psychology of Bologna University. The patients/participants provided their written informed consent to participate in this study.

## Author contributions

RB: conceptualization, methodology, investigation, data curation, writing – original draft preparation, and writing – reviewing and editing. FA: methodology, formal analysis, validation, and writing – reviewing and editing. AA: formal analysis, and reviewing and editing. GS: conceptualization, methodology, and supervision. All authors contributed to the article and approved the submitted version.

## Conflict of interest

The authors declare that the research was conducted in the absence of any commercial or financial relationships that could be construed as a potential conflict of interest.

The reviewer GM declared a past co-authorship with the author RB to the handling editor.

## Publisher’s note

All claims expressed in this article are solely those of the authors and do not necessarily represent those of their affiliated organizations, or those of the publisher, the editors and the reviewers. Any product that may be evaluated in this article, or claim that may be made by its manufacturer, is not guaranteed or endorsed by the publisher.
